# Traditionalism mediates social learning: Biases in acceptance of novel health interventions among Himba pastoralists

**DOI:** 10.1093/emph/eoag012

**Published:** 2026-06-17

**Authors:** Sean P Prall, Brooke A Scelza, Aparicio Lopes, Kassandra Schleper, Annabelle Savastio

**Affiliations:** Department of Anthropology, University of California-Los Angeles, Los Angeles, CA, USA; Department of Anthropology, University of California-Los Angeles, Los Angeles, CA, USA; OnePencil Namibia, Opuwo, Namibia; Department of Anthropology, University of California-Los Angeles, Los Angeles, CA, USA; Department of Anthropology, University of California-Los Angeles, Los Angeles, CA, USA

**Keywords:** cultural evolution, social learning, healthcare decisions, market integration, traditionalism, pastoralists

## Abstract

**Background and objectives:**

Applying social learning biases to healthcare decision-making may help explain why some people decide not to adopt health interventions. Traits like market integration and traditionalism can shape social learning biases, particularly in small scale populations with little formal education and distrust of outgroup populations. This study investigates the role of these features on social learning biases across four different health interventions among Himba pastoralists of northern Namibia.

**Methodology:**

Using vignettes from 473 participants sampled in 16 communities, we compare three social learning biases (conformity, prestige bias, parochialism) in four novel healthcare interventions. Participants completed a set of questions to estimate traditionalism and market integration. Multilevel models compared the impact of these variables on the probability of adopting the healthcare interventions.

**Results:**

Across social learning biases, conformity showed the strongest effect on likelihood of adoption, whereas we found no difference in the prestige bias condition (compared to conformity). More market integrated participants were more likely to adopt interventions, whereas more traditional participants were less likely to adopt when the outgroup advocated adoption or when most of the community didn’t adopt the intervention.

**Conclusions and implications:**

These results indicate conformity biases play a strong role in considerations about novel health interventions in indigenous communities, but also that traditional values are instrumental in shaping social learning biases. While previous focus has centered on market integration as a key predictor, we found the degree of traditionalism to be a critical and thus far underappreciated factor in mediating social learning.

## INTRODUCTION

The ability to use high fidelity social learning strategies is thought to be responsible for human cultural complexity, and a defining feature of our evolutionary success [1]. Social learning allows for the integration of new information, processes, and decisions without costly trial-and-error learning, which can lead to the speedy acquisition of fitness-enhancing information. However, learning biases can also lead to the adoption of maladaptive behaviors and perpetuate information that is out-of-date in rapidly changing environments [1]. The possibility for either a helpful or harmful outcome makes understanding the role of social learning particularly important when it comes to health interventions. For example, social learning biases can explain why the perception of peers’ vaccine status increases vaccination uptake [2, 3]. They can also help us understand why antivaccine misinformation from a layperson is more readily transmitted than medical-based views coming from a doctor [4]. These examples demonstrate the power of incorporating perspectives from cultural evolution to develop and enact health intervention strategies [5].

We still know relatively little about which social learning strategies are most effective at influencing health behaviors, or the individual factors that mediate the success of different strategies. In populations where biomedical interventions are viewed as novel, are associated with a distrusted out-group, or signal something about adherence to in-group norms or behaviors, individuals may be particularly biased in the degree to which they adhere to one strategy over another. Understanding the contextual influences of social learning mediated health-decisions needs to be better integrated into this body of work.

There are several learning biases which could benefit from this kind of contextualization. The first is prestige bias, the propensity to copy those with high-status. This model assumes that the prestige conferred to a high status individual signals something about the utility of that individual’s strategies, such that they should be a good target for imitation [6]. This has the advantage of avoiding costly evaluation of success across a population; instead one only need know who is prestigious. Prestige-biased learning is a convenient strategy, and particularly useful when learners lack experience in a particular domain or when success is difficult to assess [7, 8]. Although domain-specific expertise is often favored [9], prestigious individuals may be copied broadly, including in areas where they have no expertise, which can lead to the uptake of maladaptive behaviors. Learners also risk copying strategies that are out-of-date in a quickly changing environment if a prestigious individual is not an early adopter of new information. In the context of healthcare decisions, opinions of healthcare experts may be particularly important for their prestige and domain specific knowledge [10]. However, in our previous work we showed that individuals favored a domain-general prestigious figure (the Chief) over domain-specific ones (doctor, healer) when considering uptake of the COVID-19 vaccine [11].

As an alternative to model-based strategies like prestige bias, individuals may instead use frequency dependent approaches. Conformity bias occurs when individuals display a disproportionate tendency to copy the majority behavior in a population [12]. Models of conformity bias transmission indicate that this process can maintain group boundaries between groups and similarity within groups, and be favored by natural selection even in fluctuating environments and when information accuracy about the relevant domain is modest [12, 13]. This helps majority behaviors to remain ‘sticky,’ which could hinder the uptake of novel health interventions. On the other hand, if learners see a new behavior become popular (via exposure to an outgroup or possibly mass media exposure) conformity could help a new norm to spread. This may be particularly important when learners are uncertain about a particular domain, and when there are a number of others to learn from [14]. For example, during the COVID-19 pandemic, the propensity of a shopper to wear a mask inside a store was predicted by the proportion of others already wearing masks [15].

An additional feature to consider within this framework is the ubiquity of parochialism in humans. Parochialism is typically defined as favoritism toward the ingroup and is often associated with outgroup hostility [16, 17]. Humans have strong biases for parochial social learning (sometimes called similarity-biased social learning), where we learn preferentially from ingroup members, while simultaneously discounting information from the out-group [18, 19]. Parochial biases are particularly important when populations are diverse, and may occur even in the presence of other types of social learning strategies like conformity biases [20]. Reliance on parochialism has implicit costs, including the propensity to disregard important information when it originates in an outgroup [21, 22]. In-group preferences may be widespread, particularly when multiple ethnic groups interact, and where ethnic markers are present to signal group membership [23]. Additionally, parochialism may be magnified when there is a distrust of majority outgroups, governmental entities, or institutions. However, the degree to which parochialism limits the targets of learned information should vary depending on the benefits of that information, the degree of intragroup cooperation, and the costs and benefits of between-group competition [24]. Features of an interethnic boundary that incentivize inter-group cooperation may reduce parochial biases.

In addition to ethnic markers, there are other important sociodemographic traits that can impact social learning biases like parochialism, conformity, and prestige bias. Market integration, the shift from traditional subsistence toward reliance on market-based consumption, is one potential feature that may shape the costs and benefits of social learning. First, market integration is associated with increased levels of trust and cooperation with outgroup members [25]. This may be because market integration is associated with more interactions with strangers, leading to increased trust of outsiders [26]. Second, interactions with outgroup members can be shaped by a desire for market goods, increasing generosity toward out-group members [27]. Third, market exposure and wage labor increase the probability of adopting outgroup norms [28]. Therefore, market integration may be a particularly important component in shaping social learning strategies by increasing exposure to and trust in outgroups and their norms and lowering parochial biases.

A second feature that may mediate social learning biases is traditionalism. We define traditionalism as the degree to which one prefers the norms, morals, behaviors, and lifestyles that are historically prevalent in their particular group, while rejecting those that aren’t [29]. Traditionalism is useful for preserving adaptive patterns of behavior, particularly when the environment is static and cultural features are well suited to the local socio-ecology. But traditionalist values run the risk of preserving in-group behaviors when new technologies are more efficient [30]. In the context of social learning, traditionalism may work in the opposite direction of market integration, working to preserve in-group norms despite outgroup exposure. Traditionalism then, may preserve in-group and prestige-biases and maintain conformity bias toward existing behaviors. However, as the cultural evolutionary forces that generate traditionalism aren’t analogs to those that lead to market integration, one shouldn’t suspect a direct negative correlation.

To date, comparisons of social learning strategies are often limited to mathematical modeling, with relatively few real-world tests of social learning on healthcare decisions, particularly outside of WEIRD populations. Small scale populations, which have dense social structures, competing social influences between traditional leaders and national level politics, and vary by important axes including traditionalism and market integration, may be particularly fruitful contexts to explore these relationships and broaden the current literature. These varying features may shape the reward-based incentives of different learning strategies [31]. In this study, we aim to address the dual roles of market integration and traditionalism on social learning biases among Himba agro-pastoralists of Namibia. We compare parochialism, conformity, and prestige biases across four novel healthcare interventions. Healthcare decisions may be particularly sensitive to social learning biases in this context for several reasons. First, interventions are associated with the majority outgroup and relate to domains where few ingroup individuals have adequate information. Second, novel health interventions have costs and benefits that are often idiosyncratic and difficult to evaluate, making social learning particularly important [7, 32, 33]. Third, trial-and-error learning can be very costly in healthcare settings, making social learning valuable. To compare the influence of social learning on healthcare decisions, we rely on vignettes, which can have high external and internal validity, and are designed to reflect real world healthcare situations [34].

In many small-scale societies, social learning is likely to be complicated by mistrust of healthcare institutions. Among Himba, we have previously shown that mistrust undermines interest in vaccines and beliefs about vaccine safety [35, 36]. Additionally, Himba have a long history of marginalization by the colonial and national level governments and a low level of participation in the market economy in comparison to other ethnic groups in the region [37]. They also maintain strong ethnic markers, differentiating them from other groups. As a result, we predict parochial and conformity biases to be particularly strong. Local leaders are not likely to have domain-specific knowledge of novel healthcare interventions, which could detract from the role of prestige bias. However, we previously showed that Himba were more influenced by the Chief’s vaccine intent than other domain-specific individuals like doctors, making a priori predictions about prestige bias difficult [11]. Further, notions of prestige may be flexible, particularly in the context of increasing urbanization and exposure to market forces [38].

We further predict that uptake of novel health interventions will be moderated by market integration and traditionalism. We expect that greater market integration should increase adoption of novel outgroup interventions, as market integration should lead to greater trust and norm adoption from the outgroup, and may erode conformity to the ingroup. Conversely, since traditionalism is believed to protect ingroup norms, we predict that traditionalism should moderate social learning strategies to favor information from members of the in-group, resulting in greater ingroup conformity. Results from this study can shed light on how these moderating forces of social learning biases act in a real world population but also address how novel health information is interpreted and shaped by cultural features, with implications for public health messaging and outreach.

## METHODOLOGY

### Study population

This study was conducted in the Kunene region of Northwestern Namibia. The region is dry and arid, and characterized by high levels of inequality and low levels of development [39]. In this study, we focused on Himba pastoralists, one of the largest ethnic groups in the region. The majority of Himba in the Kunene live traditional lifestyles, residing in extended family compounds in rural areas. Himba herd cows, sheep, and goats supplemented by matrilineally inherited gardens of maize and melons. While there are some similarities with other groups in the region, including shared language, there are several strong ethnic markers that differentiate Himba. Women, in particular, have distinctive dress and hairstyles that signal ingroup membership. Himba tend to be less wealthy and less market integrated than neighboring tribes, including the closely related Herero. Himba maintain traditional leadership roles, with local and regional chiefs in cooperation with a local council of elders. Despite their cultural resilience, Himba communities are undergoing rapid cultural change due to exposure to outgroups, but also as a response to recent drought and loss of livestock which has necessitated an influx of market-bought foods [40, 41].

### Data collection

**Figure 1:**
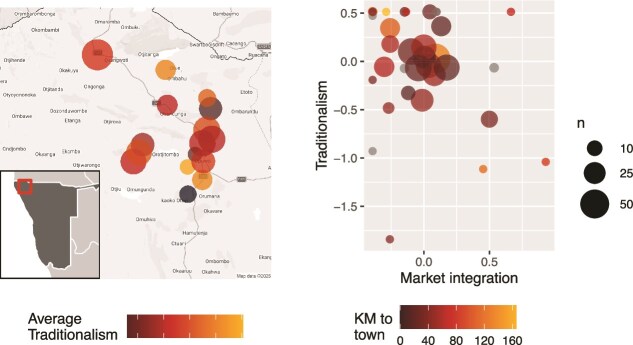
Map of sampled communities (left) and scatterplot showing the relationship between traditionalism, market integration, and distance to town (right). For map of sampled communities, inset map indicates sampling area. For each community, average traditionalism score is shown in color. Communities with fewer than five participants or with unknown GPS locations are not shown, and point size indicates sample size. Scatterplot shows average level of traditionalism and market integration scores, with color indicating kilometer distance to local capital. All resident villages shown, irrespective of sample size. Gray points are those where participant residence location is unknown. For visual purposes one outlier is excluded.

We conducted a cross-sectional survey of Himba adults sampled in 16 Himba communities. Sampling locations were opportunistically chosen from known Himba communities spanning a rural–urban gradient, but accessible by 4WD vehicle. We also sampled in the regional capital of Opuwo, where a small minority of Himba reside, recruiting from local markets and public spaces. A map of participant residence locations is shown in Fig. 1. All data was collected between July and October of 2025, and all Himba adults in the focal villages were eligible to participate. Interviews were conducted in private in *Otjiherero,* primarily by a local research team, using the KoboCollect program on tablet computers [42]. This work was approved by the UCLA Institutional Review Board (IRB #10-000238).

### Predictors

To measure market integration, participants were asked a set of questions regarding dwelling materials, latrine type, any formal education, and whether they had worked for cash in the last year. To estimate degree of traditionalism participants were shown sets of digital illustrations of figures in western or traditional Himba attire. These images were used as prompts for the following questions: ‘who would prefer your [son/daughter] to marry?,’ ‘who would you rather have your [son/daughter] look like?’ and ‘which man do you respect more?’ For each question, participants either chose either a figure representing a traditional Himba, or a person in western dress [see Ref. 41 for example]. For each of these measures, binary outcomes were coded and then a single latent factor estimated from both traditionalism and market integration using latent trait models via the *mirt* package [43]. Loadings and additional model details are shown in the supplemental materials document.

### Social learning vignettes

Each participant was randomly assigned one of six sets of vignette questions (see Table 1 for an example). Each set contained six questions across four novel healthcare interventions (vaccination, water purification, micronutrient supplementation, and household mosquito spraying) and three social learning frames (parochialism, conformity, and prestige). For each vignette, participants were presented with a scenario and asked to choose whether they wanted to adopt the novel health intervention or not (Table S2).

**Table 1:** Example of vignette sampling strategy.

	Parochialism	Conformity	Prestige
Vaccination	✓ (+)	✓ (−)	✓ (+)
Water Purification	✓ (−)		
Micronutrient Supplementation		✓ (+)	
Household Mosquito Spraying			✓ (−)

This table shows an example of the set of six vignettes one participant would receive. Each participant was given six vignettes: three vaccine vignettes, one for each social learning frame (parochialism, conformity, prestige), and one vignette for each of the other three interventions. Positively and negatively valenced versions of each vignette were created and each participant received three positively valenced and three negatively valenced vignettes.

Participants were shown a set of story cards depicting each scenario for clarity (Figs S1–S3). As we were principally interested in vaccine decision-making, three of these questions focused on vaccine decisions, while the other three presented vignettes about the other public health interventions. For example, the vaccine vignette described a novel COVID-19-like virus and a vaccine recommended by the government to counter it. Each intervention was linked to three social learning frames where participants were given a description about who had decided to adopt the intervention. In the parochialism condition, a Himba man received the vaccine and an Owambo (majority outgroup) man did not, or vice-versa (for example: ‘Kemuu, who is Himba says that people should get the vaccine. John, who is Owambo, says that people should not get the vaccine. What would you do?’). In the conformity condition, the question described how most people in the community had or had not decided to get the vaccine. In the prestige condition, the question described how either the chief (local village leader) or the community had gotten the vaccine while the other had not. The vignettes each participant saw were split between being positively and negatively valenced. In positive vignettes the chief/majority/ingroup enacted the intervention, while in negative vignettes they did not. When possible, we opportunistically asked participants to explain their decision-making. All vignettes and more information about the sampling framework is available in the supplementary materials document.

### Analysis

A set of Bayesian multilevel models were used to predict probability of adoption of the health intervention via the *brms* package in R using RStudio [44–46]. For each social learning frame, the probability of the intervention was predicted by gender, age, the intervention advocate (for example, ingroup or outgroup in the parochialism frame), traditionalism estimate, and market integration estimate. We included interaction effects between traditionalism, market integration, and intervention advocate since we predicted that these variables should be mediated by the source of information. As model pools different health intervention domains, these predictors varied by each of the four domains via varying slopes. Additional varying intercepts to correct for repeated measures by individual and by village of residence. All models used regularizing priors. Where relevant we report the probability of posterior distributions falling above or below zero (pr[β > 0] or pr[β < 0] respectively). Additional packages for data cleaning, modeling, and visualization include *tidyverse, tidybayes, patchwork, janitor,* and *ggmap* [47–51]. Additional modeling details are shown in the supplementary materials.

## RESULTS

We collected 2827 responses to vignettes from 473 individuals (Table S1). Respondents resided in 36 different communities, and as far away from town as 165 km (average distance is 47.3 km). Respondents age ranged from 16 to 95, with an average of 36.4 years, and 63.2% of respondents were women. Latent trait models for market integration produced a single factor of moderate strength with positive loadings from 0.37 to 0.83 (Table S3). For traditionalism, latent trait models produced a single strong factor with positive loadings from 0.78 to 0.93 (Table S4, Figure S4). At the individual level, market integration and traditionalism factors were weakly negatively associated (Pearson’s $\mathrm{\rho}$ = −0.16, 95% CI = −0.25 to −0.07), although at the community level, where communities had at least five respondents, these variables were not consistently associated (Pearson’s $\mathrm{\rho}$= −0.39, 95% CI = −0.74 to 0.12). The relationship between community level averages for traditionalism and market integration are shown in Fig. 1.

**Figure 2:**
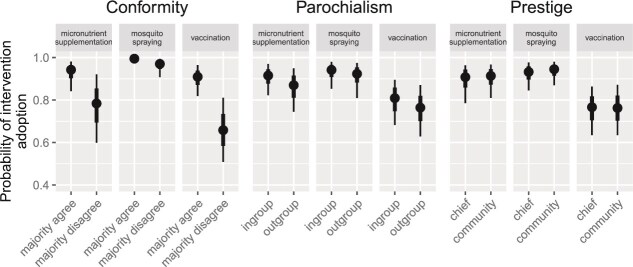
Posterior predictions of vignettes by domain and condition. Point and whisker plots represent posterior median, and 66% (thick line) and 95% (think line) credible intervals. Water purification results excluded for space, as respondents chose to adopt this intervention in 100% of vignettes.

Respondents were generally supportive of the novel health interventions. Across social learning frames, respondents supported micronutrient supplementation in 76.5% of vignettes, mosquito spraying in 86.6% of vignettes, vaccination in 68% of vignettes, and water purification in 100% of vignettes, regardless of the type of vignette or the party advocating adoption. Model results are shown in Fig. 2. The conformity condition showed the strongest effect by advocate, such that participants were more likely to adopt the intervention when the community had adopted it, and less likely to adopt when they had not (pr[β < 0] = 99%, Fig. S5). Conversely, we found very small differences in the parochialism condition between when the advocate was in the in-group versus the out-group. While the probability of adoption was generally higher when advocated for by an in-group member, these differences were not meaningful (pr[β < 0] = 84.6%, Fig. S6). We found no consistent differences in the prestige condition comparing the chief against the community (pr[β < 0] = 40.9%, Fig. S7). Differences in posterior predictions are shown in Fig. S12. Age and gender effects were relatively minor (Fig. S10 and S11)

**Figure 3:**
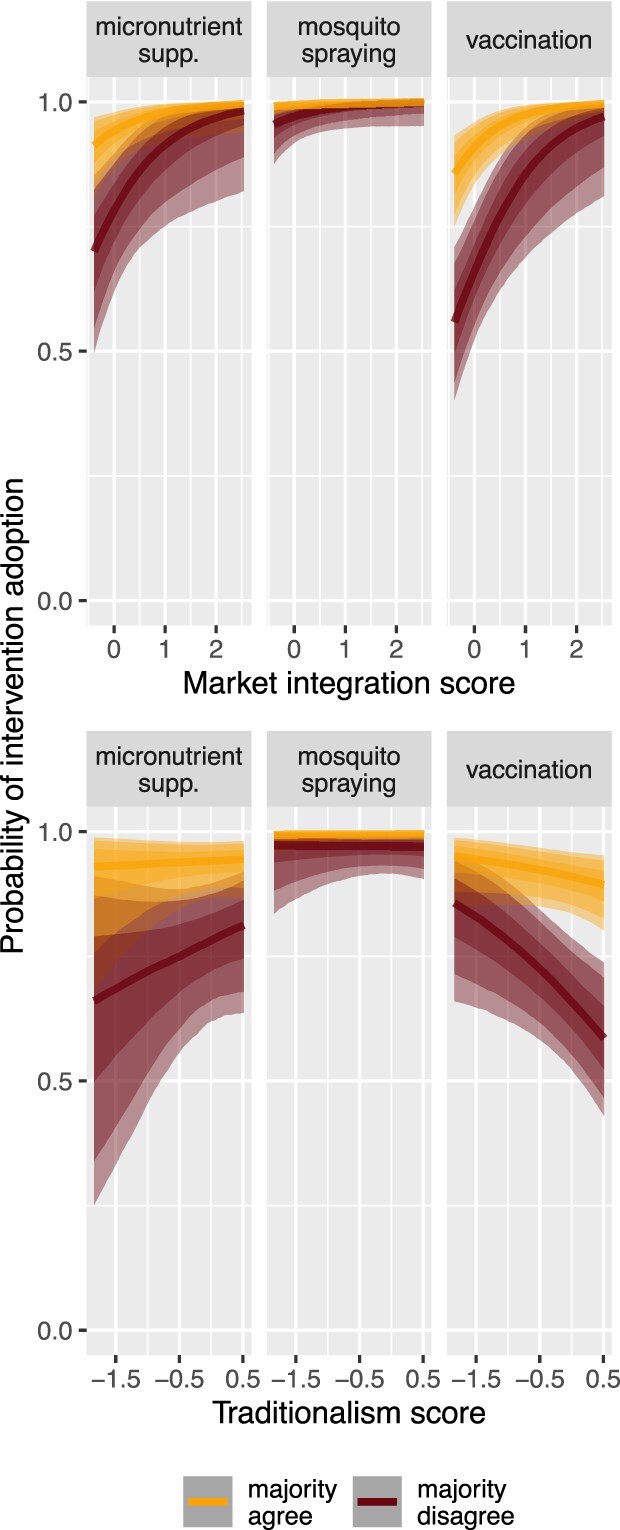
Posterior predictions for conformity condition by level of market integration and traditionalism. Shading represents 50%, 80%, and 90% prediction intervals. Water purification results excluded for space, as respondents chose to adopt this intervention in 100% of vignettes. Market integration was associated with an increased probability of intervention adoption, while associations with traditionalism varied by intervention type and advocate. For example, more traditional respondents were less likely to adopt the vaccine intervention when the majority of the community chose not to adopt.

**Figure 4:**
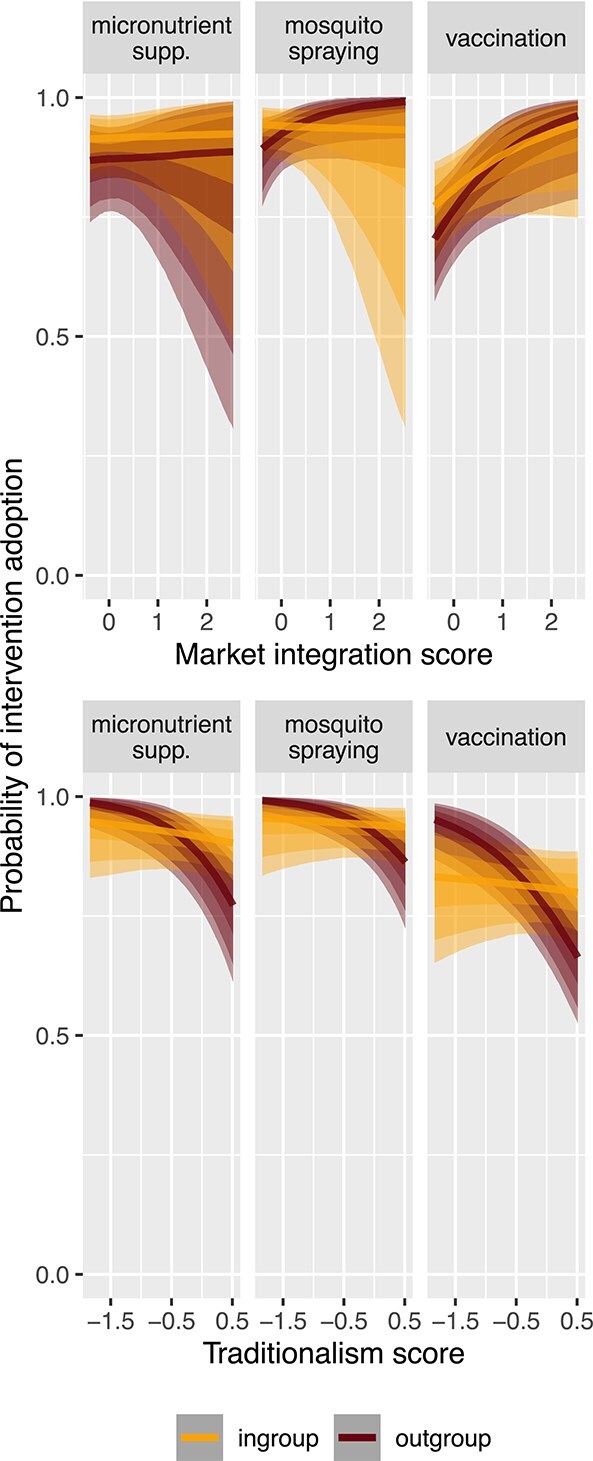
Posterior predictions for parochialism condition by level of market integration and traditionalism. Shading represents 50%, 80%, and 90% prediction intervals. Water purification results excluded for space, as respondents chose to adopt this intervention in 100% of vignettes. Market integration had little impact on intervention adoption in this condition. The impact of traditionalism varied by intervention advocate. When intervention was advocated for by the ingroup, traditionalism had no association with the probability of intervention adoption. However, when traditionalism was advocated for by the outgroup, more traditional respondents were less likely to adopt the intervention across three intervention types.

The effects of market integration and traditionalism showed inconsistent effects, mediated by experimental frame and intervention in question. In the both the conformity and prestige frames, market integration was associated with increased intervention adoption (pr[β > 0] = 97.2% for both), but not in the parochialism frame (pr[β > 0] = 64.8%, Fig. 3). This effect was particularly strong for the vaccination intervention. However, market integration did not show interactions with the intervention advocate in question for each social learning frame. Traditionalism, on the other hand, was strongly dependent on the adoption advocate in question across social learning frames. For the conformity frame, traditionalism tended to decrease the probability of vaccination, but only when the community did not adopt the intervention. In other words, more traditional respondents were more conformist when the community didn’t adopt vaccination, but traditionalism was not associated with adoption when the community did adopt the vaccination. However, in the micronutrient supplementation condition, more traditional respondents were more likely to adopt the intervention when the community didn’t adopt. Traditionalism played a stronger mediating role in the parochialism social learning frame. More traditional respondents were less likely to adopt three of four interventions when it was advocated by the outgroup, but there was no association when it was advocated by the ingroup (Fig. 4). This effect was strongest with vaccination. In the prestige condition, we saw no mediating relationship between traditionalism and the influence of chief versus community on intervention uptake (Fig. S9).

## DISCUSSION

In this study, we compared the role of market integration and traditionalism on social learning biases across four novel health interventions. Our results suggest that the population is highly supportive of health interventions, with the majority choosing to adopt, irrespective of the social learning frame or the agent advocating adoption. When comparing the three social learning frames, we found conformity bias had the strongest impact on adoption of the interventions. Conversely, we found only slight differences in the parochialism condition, comparing the influence of ingroup versus outgroup members, and no difference in the prestige condition comparing the chief to the community.

### Conformity, but not prestige or parochialism, predicts intervention uptake

Previous research has shown conformity bias is linked to health behaviors including drinking, diet, vaccination decisions, mask-wearing, and even birth practices [2, 15, 52–54]. Our results align with these findings. Conformity biased transmission is believed to be a particularly important feature of social learning when learners are uncertain about the domain, when the domain is difficult, and when asocial learning is costly or challenging [14]. We found the conformity effect to be strongest in the vaccination vignette, which was centered on a vaccine for a novel COVID-19-like disease. Previously, we showed both high uncertainty about the COVID-19 vaccine, and lack of knowledge about both COVID-19 and the vaccine [55]. This may explain why the similar, hypothetical vaccine profiled here triggered the use of conformity bias.

Surprisingly, we found a propensity for participants to adopt the water purification and mosquito spraying interventions, even in vignettes where the community rejected these interventions. However, these interventions may have been particularly desirable during the period when this study took place, leading to a greater propensity to accept, regardless of community support. Himba traditionally rely on hand-dug sandwells or boreholes for water, although these are sometimes unreliable or dangerously unclean [56]. The Kunene experiences periodic droughts, including a recent severe decade-long drought, and access to water for gardens, livestock, and for household use is a major concern for Himba [57, 58]. Simultaneously, Namibia experienced a major malaria outbreak in during the 2024–2025 rainy season [59]. While not all Himba recognize mosquitos as the source of malaria, it is a disease with a long history in the area and specific cultural models of illness and numerous traditional treatments [60]. Additionally, water purification tablets, and to a lesser extent mosquito spraying, are interventions that some communities are familiar with, which may make the need to rely on social learning less critical for these domains.

We found very weak effects of parochialism and prestige in these vignettes. Anecdotally, many participants agreed to the intervention for idiosyncratic health reasons and did not reflect on the source of information. For participants who chose to adopt the intervention advocated by the outgroup member, many raised the prospect that the Owambo (out-group) person mentioned in the vignette may be more educated and knowledgeable about that particular domain. For example, one 57-year-old woman explained her choice this way: ‘*This one* [Himba] *is not educated, the other* [Owambo] *is educated. I can see from the clothes. How can I follow the one in the skins?*’ In the prestige condition, we asked respondents to compare the influence of the chief to that of the community. Results indicate the chief had little influence compared to community, but this finding may be outweighed by the preference for conformity. Anecdotally, some participants who ignored the decision of the chief emphasized that it is better to follow the community, or as one respondent noted: ‘*I would not follow one person, I would go where the majority goes*.’ Conversely, when siding with the chief for the intervention, many participants mentioned the importance of following the chief or raised health specific information. For example, in agreeing to mosquito spraying as advocated by the chief, one participant explained; ‘*because I want to prevent the mosquito, I won’t listen to the stubborn people*.’ In a previous qualitative study of COVID-19 vaccine decision making, we found that while local leaders can influence the community, particularly when it comes to interfacing with healthcare outreach, respondents emphasized that vaccination was ultimately an individual decision [55].

### Traditionalism moderates learning biases

We measured both market integration and traditionalism to assess their roles in mediating healthcare decisions. In some cases, market integration has been used as a proxy measure of traditionalism, assuming that individuals who are more market integrated must be less traditional in their norms and beliefs. Our results suggest that conflating these measures is a mistake as the two measures were only very weakly correlated at an individual level, and unrelated at the community level. Whereas market integration may be associated with exposure to and trust in outgroups, traditionalism is likely to be associated with the maintenance of ingroup specific beliefs and behaviors.

In accordance with our predictions, market integration was generally associated with increased intervention adoption. This was particularly true for vaccination, which was more likely to be endorsed in more market integrated respondents across all social learning frames. This corresponds with our previous work in the area showing that urban and peri-urban residents were more likely to report receiving the COVID-19 vaccine [36]. We also previously found with Himba that market integration is associated with greater internal locus of control, indicating a higher belief in self-efficacy, and suggesting they may be more comfortable accepting novel health behaviors [61]. Kunene residents with more education and those living closer to town also tend to have a more healthcare knowledge [55, 60]. However, contrary to our prediction, our results do not indicate that more market integrated individuals are more likely to trust outgroup sources. In the parochialism frame, market integration did not mediate the probability of adoption when the information advocate was a member of a different ethnic group. Although interaction with outgroup members does not have an effect, more market integrated people likely have more exposure to health messaging through media and have more frequent engagement with the healthcare system. Interaction with domain specific health sources like these might matter more than interactions with outgroup members at large.

In line with our predictions, traditionalism was linked with favoring in-group sources of information. In the parochialism frame, more traditional respondents were less likely to adopt three of four interventions when they were advocated by the outgroup. Similarly, traditionalism eroded support for vaccination in the conformity frame when the community was not adopting the intervention. These results suggest that traditionalism preserves in-group and conformist social learning. More traditional populations may be resistant to novel healthcare interventions, particularly when they are associated with sources of information from the outgroup. In this study, these associations occur independent of market integration and imply that traditionalism is a separate feature of the population that can be measured to explain resistance toward outgroup messaging.

### Limitations and future directions

Our study is not without limitations. We attempted to construct vignettes across different domains of healthcare to sample domains where we expected varying opinions. However, our study population was largely in favor of all the interventions, particularly in domains like water purification, limiting variability in our outcome variables. This may be due, in part, to social desirability bias. However, a majority of these data were collected by a Namibian research team, with American researchers working with them for the first phase of data collection. If present, this bias could have led to a higher ceiling for stated preferences than we would see in measures of adoption for these interventions.

Most of the communities sampled had some familiarity, or were at least aware of, all the interventions used in the vignettes, which may undermine the need to use social learning in making decisions. However, we opted for interventions that were familiar for several reasons. First, a previously established high level of medical mistrust in these communities may have led to overinflated rejection of novel interventions [35, 36]. Second, explaining novel interventions would have been time-intensive and increased the possibility of misinterpretation or error. Finally, the funding for this project was focused mainly on vaccination decisions, necessitating inclusion of a vaccine-related intervention. Future research which uses vignettes that vary in their novelty may better assess the importance of familiarity in decision making.

There were also some limitations to the way we constructed our study design and social learning conditions. In the prestige learning frame, we compare a prestigious figure (Chief) to the community, rather than testing the strength of prestige bias on its own. This was done purposefully, as we had previously looked at prestige bias on a similar social learning intervention related to vaccines, and shown evidence of its effect [11]. Our parochialism frame references a specific outgroup, but may not generalize more broadly, and references to other outgroups may yield different results. Our conformity condition captures a tendency to follow the majority, but doesn’t capture the frequency-dependent tendency to disproportionately adopt an intervention. To do that, we would have needed data on how many people in the community previously adopted these interventions and that data was not available as a reference. Lastly, we only compare three social learning frames, but there are several others that may be worthy of inclusion in future studies, alongside additional cognitive, behavioral and structural factors that contribute to decision making surrounding novel health interventions [62, 63].

Cultural evolutionary theory holds promise for understanding behavior and decision making when applied to health. In this study, we sought to examine how two sociocultural features, market integration and traditionalism, impact social learning biases to novel healthcare interventions. We find strong learning biases for conformity, but weak biases for prestige and parochialism. However, these biases were primarily shaped by degree of traditionalism, which acts to preserve ingroup sources of information. These findings suggest that social learning strategies are sensitive to local contextual factors [64]. In particular, traditionalism may shape the rewards to different social learning strategies, and where there are strong norms of behavior in place, traditionalism may signal an adherence to those behaviors [31]. Notably, in contrast with other studies, which implicitly or explicitly include traditionalism within measures of market integration, we found associations with traditionalism occurred independent of market integration. This suggests that traditionalism should be studied as a separate feature of the population and has its own important effects on healthcare decisions. Applying social learning to healthcare decision-making, particularly in places where healthcare needs collide with epistemic distrust of outgroups and healthcare institutions, can help both evolutionary social scientists and public health professionals better understand how these important decisions are made.

## Supplementary Material

Supplemental_Materials_eoag012

## Data Availability

Data and R code supporting this manuscript is available at: https://osf.io/8csxd/.
