# Supplementary material for: Traditionalism mediates social learning: Biases in acceptance of novel health interventions among Himba pastoralists
Source: Evol Med Public Health. 2026 Jun 17;14(1):1–12. doi: 10.1093/emph/eoag012 (PMC13366536; doi:10.1093/emph/eoag012)
Supplement: Supplemental_Materials_eoag012 [file supplemental_materials_eoag012.pdf]

Supplemental Information

Traditionalism mediates the social learning: Biases in acceptance of novel health interventions among Himba pastoralists

Prall et al., in *Evolution, Medicine, and Public Health*

Additonal details on the social learning vignettes

Vignette counts

Participants randomly received one of six sets of vignettes. Each included three questions about vaccines across the three social learning frames, followed by one each of the other health intervention domains, randomly assigned to a social learning frame. Question valence was also randomized. As a result, we collected 1412 complete vaccination vignettes, 473 micronutrient supplementation and mosquito spraying vignettes, and 469 complete water purification vignettes. Vignette counts by intervention and social learning frame are shown below. Note that due to a randomization error during the first few days of data collection, counts depart slightly from what would be expected.

Table 1: Counts of vignettes by social learning frame and intervention type

intervention	conformity	parochialism	prestige
micronutrient supplementation	146	183	144
mosquito spraying	144	146	183
vaccination	468	472	472
water purification	181	144	144

Vignette examples

The **vaccination vignette** began as follows: "*There is an outbreak of a new disease that is spreading throughout the world, including in Namibia. Many people are getting very sick and some have died. There is now a vaccine for the disease and some people are recommending getting it because it will help prevent spread of the disease, while others are not getting it because they fear it might not be safe.*"

Next, participants were randomly assigned one of the following for the parochialism condition:

- Parochialism with negative valence: "*John, who is Owambo says that people should get the vaccine. Kemuu, who is Himba, says that people should not get the vaccine. What would you do?*"
- Parochialism with positive valence: "*Kemuu, who is Himba says that people should get the vaccine. John, who is Owambo, says that people should not get the vaccine. What would you do?*"

Next, participants were assigned one of the following from the conformity condition:

- Conformity with negative valence: "*Most people in your community have decided not to get the vaccine. What would you do?*"
- Conformity with positive valence: "*Most people in your community have decided to get the vaccine. What would you do?*"

Lastly, participants were assigned one of the following prestige bias conditions:

- Prestige bias with negative valence: "*Most people in your community have decided to get the vaccine, but the Chief has decided not to get the vaccine. What would you do?*"
- Prestige bias with positive valence: "*Most people in your community have decided not to get the vaccine, but the Chief has gotten vaccinated. What would you do?*"

Following these three vaccine questions, participants received one each of the remaining health intervention vignettes, where social learning frame and valence was randomly assigned. For the **mosquito spraying vignette** participants were first told the following: "*Malaria rates in Namibia have been higher than normal recently. The government is recommending that people get their houses sprayed to kill mosquitos. Some people are worried that the spraying is bad for their health.*" After this setup statement, they then received the question as described above, although the placeholder names were changed in the parochialism frame for each health outcome. For the **micronutrient supplementation vignette**, participants were told the following: "*To help children stay healthy, nurses in Namibia recommend that mothers add a special powder to maizemeal. The nurses say that the powder will help make the children strong and they will get sick less. Other people worry that the powder might be dangerous for the children.*" For the **water purification vignette** participants were told the following: "*To keep water safe and clean the government is recommending that people add tablets to their water containers. The tablets help eliminate diseases that are in dirty water. But some people worry that the tablets could make people sick.*" The table below shows a summary of question sets used in this study.

Table 2: Example question set

question	domain	frame	valence
1	vaccination	parochialism	+/-
2	vaccination	conformity	+/-
3	vaccination	prestige	+/-
4	mosquito spraying	randomly selected	+/-
5	micronutrient supplementation	randomly selected	+/-
6	water purification tablets	randomly selected	+/-

Example images

In addition to the question prompts described above, participants were shown a corresponding set of images for each. Images were AI generated, and used to help explain the particular vignette setup. For example, Figure S1 shows all four images used for the health intervention domain setup statements, to aid in explanation of these interventions. Figure S2 shows the cards used in the vaccination vignette for parochailism with positive valence. Figure S4 shows the cards used in the mosquito spraying vignette for prestige bias with negative valence.

Figure S1: Vignette setup images. These images were shown to participants as part if the initial description of each health intervention vignette to aid understanding.

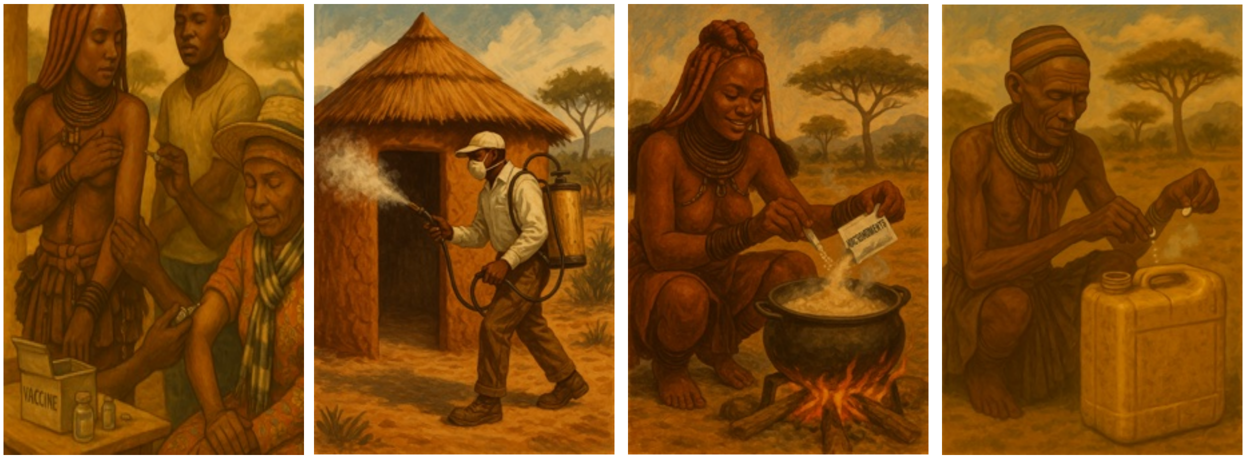

Figure S2: Parochialism and vaccination vignette example images. These images were shown to participants in the positively valenced parochialism/vaccination condition.

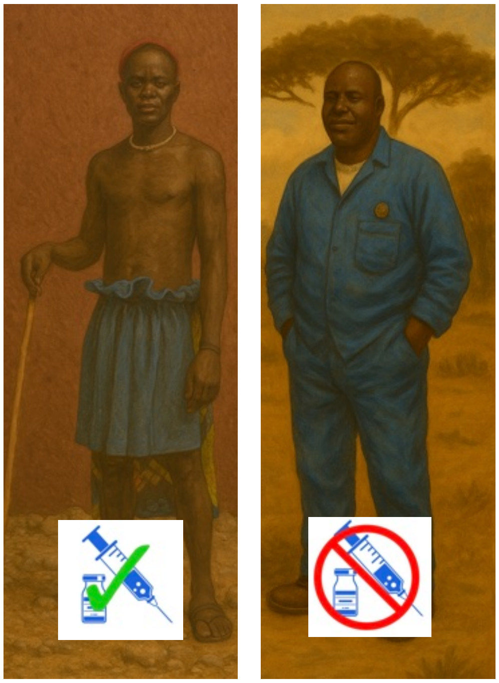

Figure S3: Prestige bias and mosquito spraying example images. These images were shown to participants in the mosquito household spraying prestige condition.

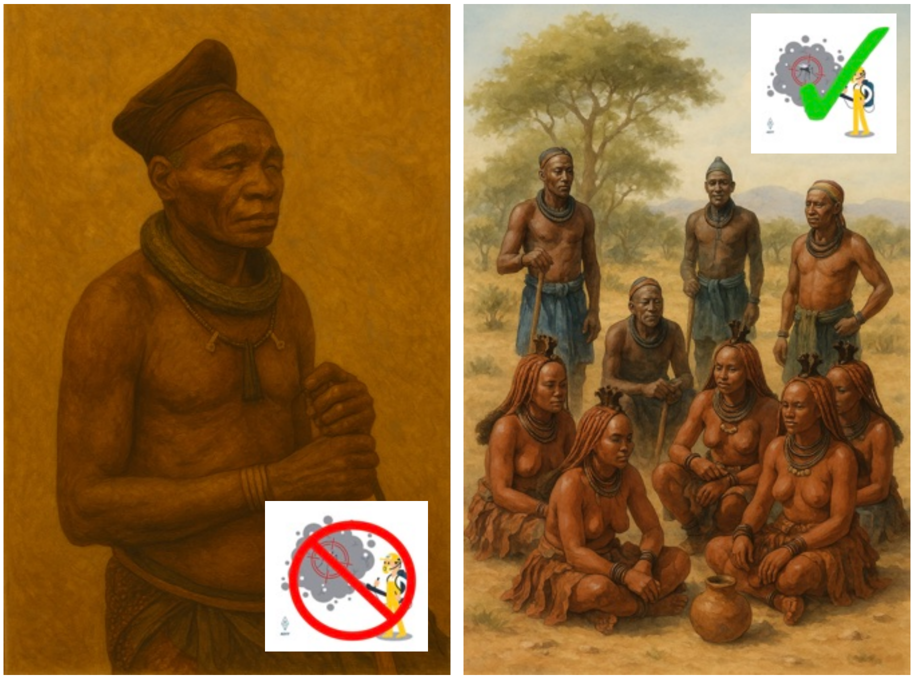

Latent trait model for market integration

Market integration was estimated via a set of binary questions, including roof type, latrine type, cash labor participation in the past year, and any formal education. Other variables were discarded due to strong correlations with other variables, weak loadings, or lack of variability in responses. The *mirt* package in R (Chalmers 2012) was used to estimate a single latent market integration scale. Testing of various models indicated some potential for a multidimensional structure. However, bifactor models increased complexity without improvement in information criteria values, so a unidimensional model was used. Results indicate a general latent factor best explained the majority of the variance (39%). As a result, we rely on the single latent factor derived from these four binary predictors in our analysis for use in later models. Model loadings are shown below.

Table 3: Loadings for the latent trait model for market integration

variable	F1	h2
roof type	0.369	0.136
latrine type	0.832	0.693
worked for cash in the last year	0.621	0.386
any formal education	0.581	0.338

Latent trait model for traditionalism

Traditionalism was estimated via a set of five binary. Participants were shown sets of digital illustrations of figures in western or traditional Himba attire which were used as prompts for three questions: “who would prefer your [son/daughter] to marry?,” “who would you rather have your [son/daughter] look like?,” and “which man do you respect more?” For each question, participants either chose either a figure representing a traditional Himba, or a person in western dress. Example illustrations are shown in Scelza et al. (2019). We also collected data on participant dress (traditional or western), and whether they had their incisors

removed (a traditional practice). We again used *mirt* to estimate a single latent trait from traditionalism. Dress and incisor ablation showed weak and inconsistent loadings in initial models and had little impact on shared variance. As a result these were excluded. The final model explained 83% of variance and produced a single latent factor for use in multi-level models. Loadings are shown below. We also plot the relationship between the estimated latent trait for traditionalism and a raw score computed by adding up relevant binary variables. These two scales show a strong positive correlation.

Table 4: Loadings for the latent trait model for traditionalism

variable	F1	h2
daughter marry	0.930	0.864
daughter look	0.920	0.846
son marry	0.943	0.890
son look	0.965	0.932
respect	0.780	0.608

Figure S4: Comparison between latent trait and addive traditionalism score

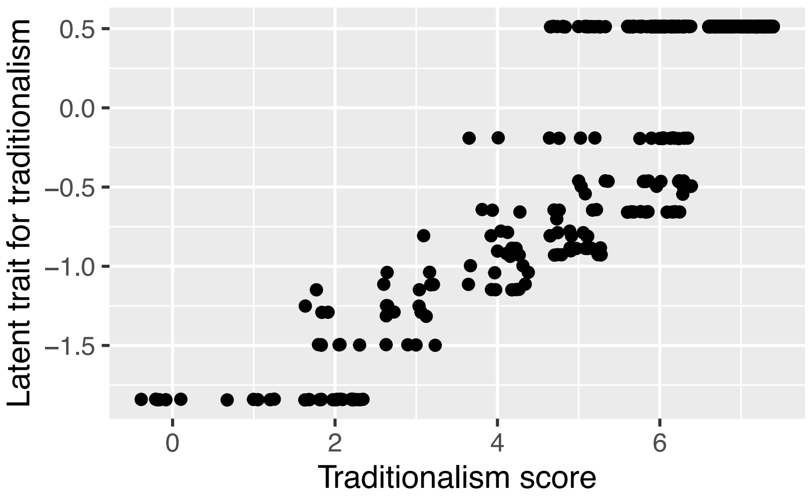

Modeling details

To estimate the probability of intervention adoption for each social learning frame (three separate models), a Bernoulli multilevel model was defined below was used, where *IA* represents the intervention advocate, *trad* represents the estimated traditionalism score, and *mi* represents the estimated market integration score. Varying intercepts for the health intervention domain, as well as community and individual respondent, were included. Varying slopes for all predictors by domain were also included. For the predictors described and plotted below, reference category for gender is female. For information advocate, across models using different social learning frames, reference category is ingroup, community, and chief for the parochialsim, conformity, and prestige models respectively.

$$
\begin{aligned}
\textit{intervention adopted} &\sim \text{Bernoulli}(p) \\
\text{logit}(p) &= \alpha + \alpha_{location} + \alpha_{id} + \alpha_{domain} \\
&\quad + (\beta_{gender} + \beta_{gender[\text{domain}]}) \cdot \text{gender} \\
&\quad + (\beta_{age} + \beta_{age[\text{domain}]}) \cdot \text{age} \\
&\quad + (\beta_{ia} + \beta_{ia[\text{domain}]}) \cdot \text{ia} \\
&\quad + (\beta_{trad} + \beta_{trad[\text{domain}]}) \cdot \text{trad} \\
&\quad + (\beta_{mi} + \beta_{mi[\text{domain}]}) \cdot \text{mi} \\
&\quad + (\beta_{ia \cdot trad} + \beta_{ia \cdot trad[\text{domain}]}) \cdot (\text{trad} \cdot \text{ia}) \\
&\quad + (\beta_{ia \cdot mi} + \beta_{ia \cdot mi[\text{domain}]}) \cdot (\text{mi} \cdot \text{ia})
\end{aligned}
$$

For these models, regularizing priors were used for all intercepts ($\alpha \sim \text{Normal}[0, 1]$), predictors ($\beta \sim \text{Normal}[0, 1]$), variance parameters ($\sigma \sim \text{Exponential}[1]$), and correlation parameters ($\rho \sim LKJ[2]$). Model convergence was assessed by inspecting $\hat{R}$ values. All models used 4000 iterations, half of which were warm-up, and run on four chains, and the target average acceptance probability (adapt_delta) was set to 99%. Full model posteriors are shown in figures S5 to S7 below. All modeling was done using the *brms* package (Bürkner 2015) in R (version 4.5.2).

Figure S5: Conformity model posterior distributions. Abbreviations and coefficients are defined in the modeling details section.

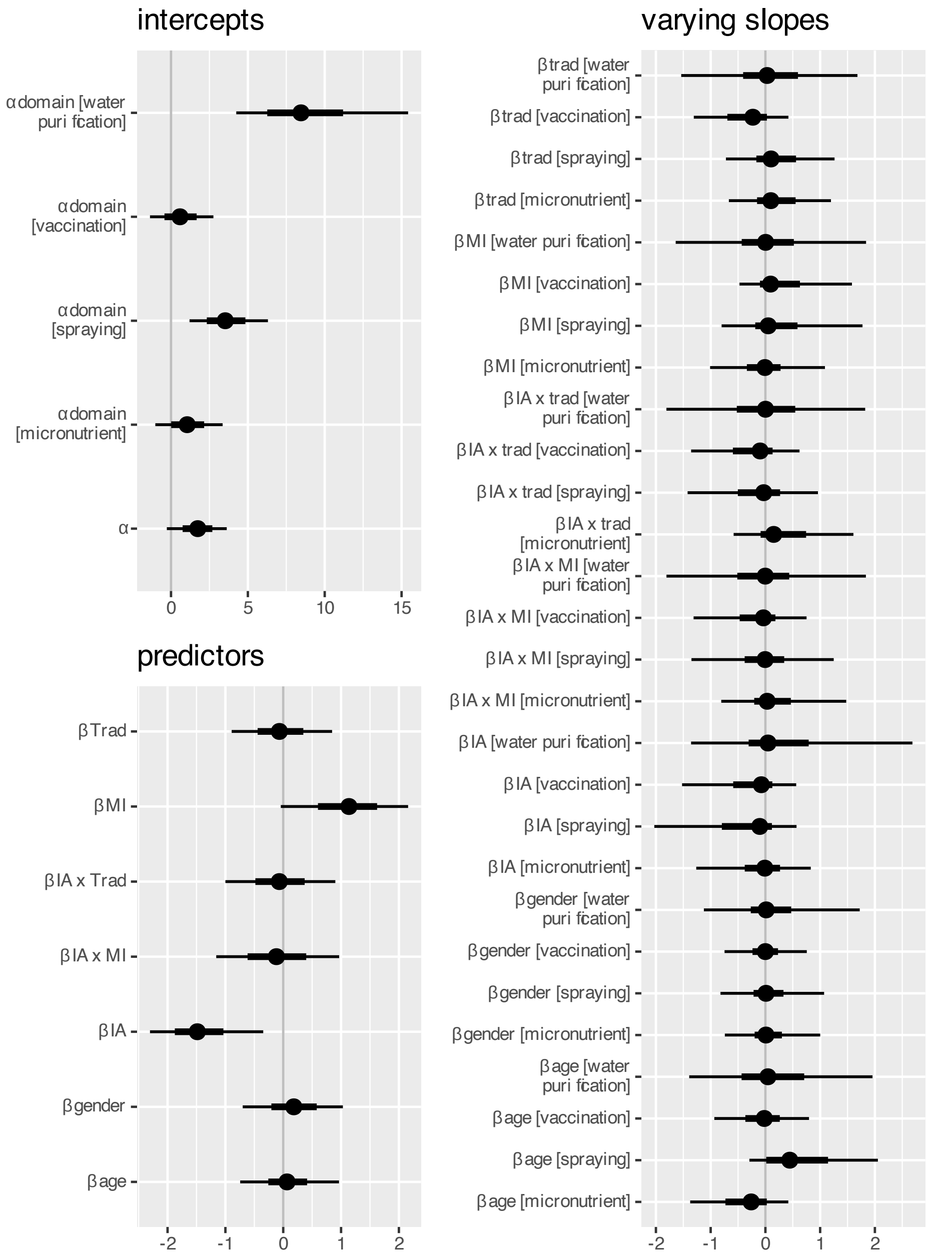

Figure S6: Parochialism model posterior distributions. Abbreviations and coefficients are defined in the modeling details section.

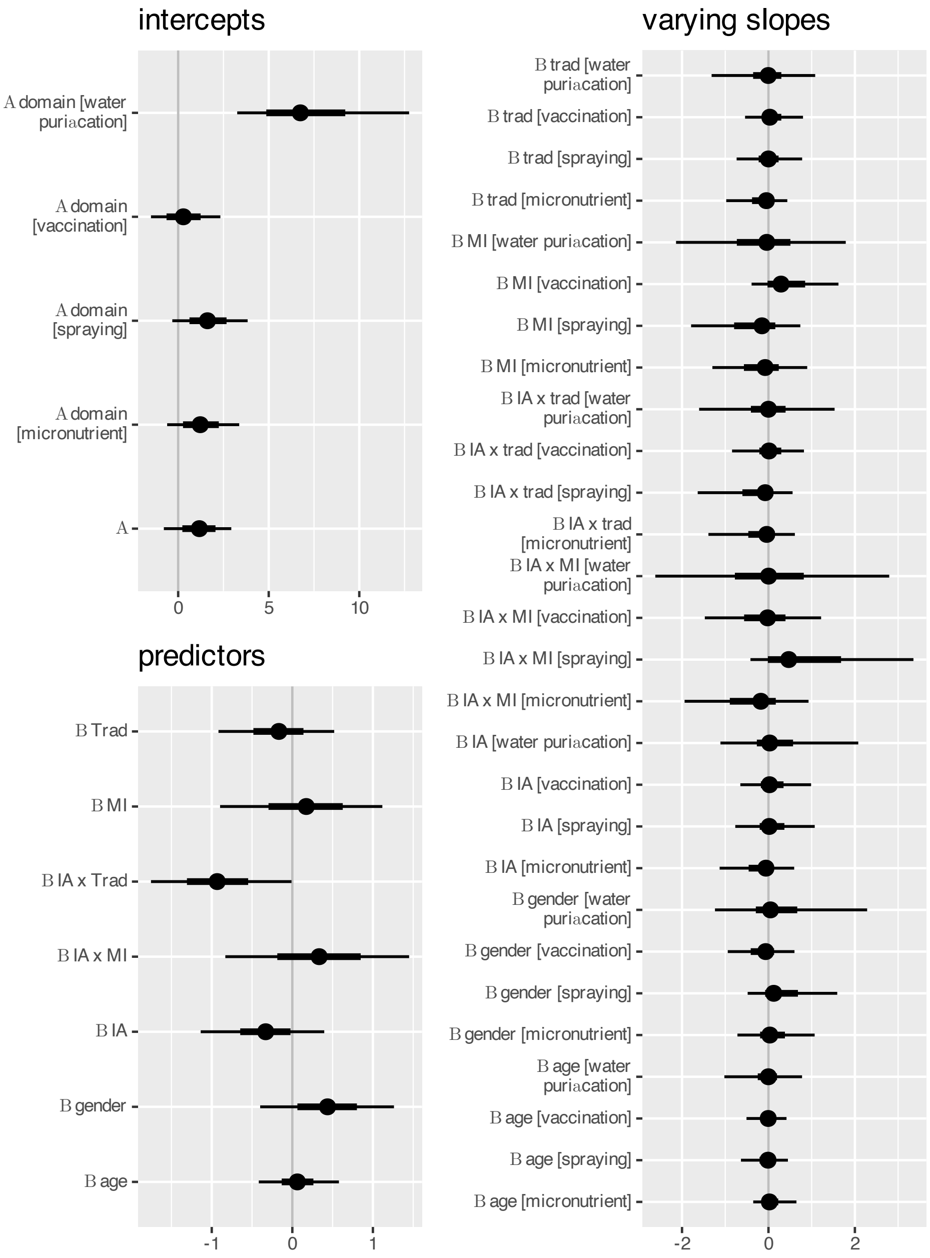

Figure S7: Prestige model posterior distributions. Abbreviations and coefficients are defined in the modeling details section.

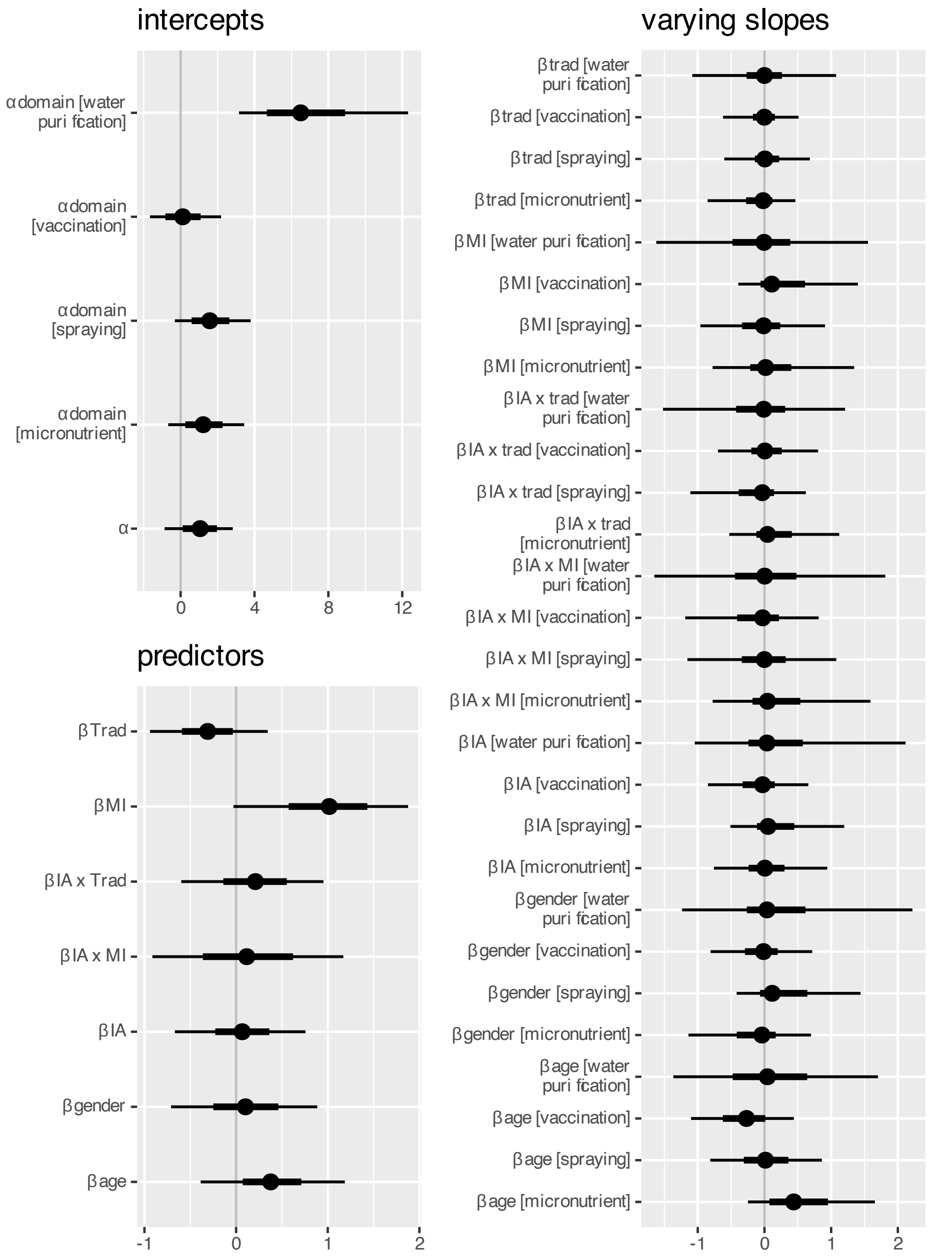

Additional plots

Figure S8: Community map by level of market integration

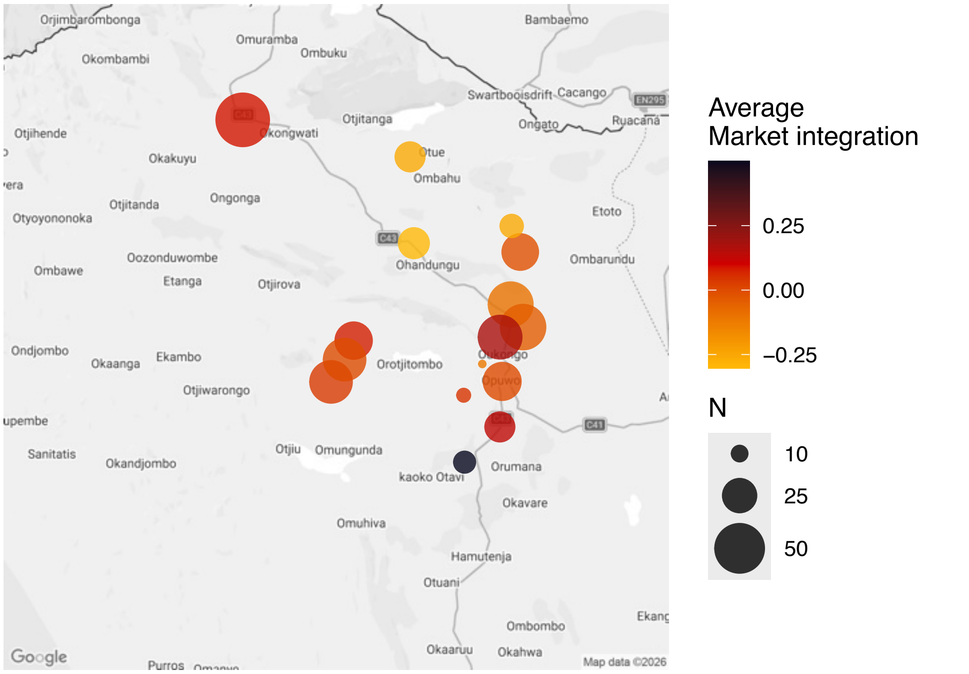

Figure S9: Posterior predictions for the prestige condition by level of market integration and traditionalism. Shading represents 50%, 80%, and 90% prediction intervals. Other social learning frame predictions are shown in the main text.

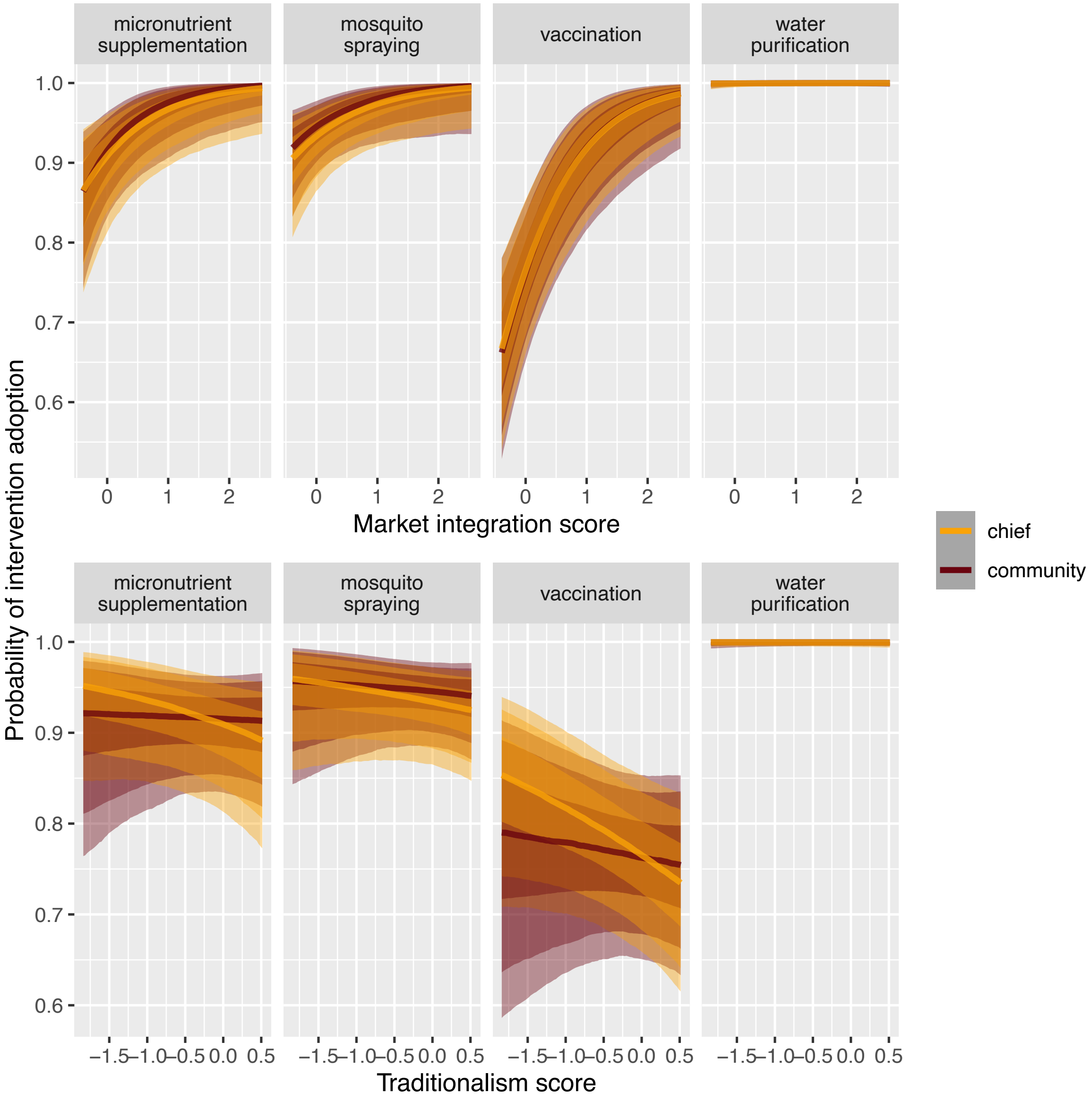

Figure S10: Posterior predictions for age on intervention uptake by condition Shading represents 50%, 80%, and 90% prediction intervals.

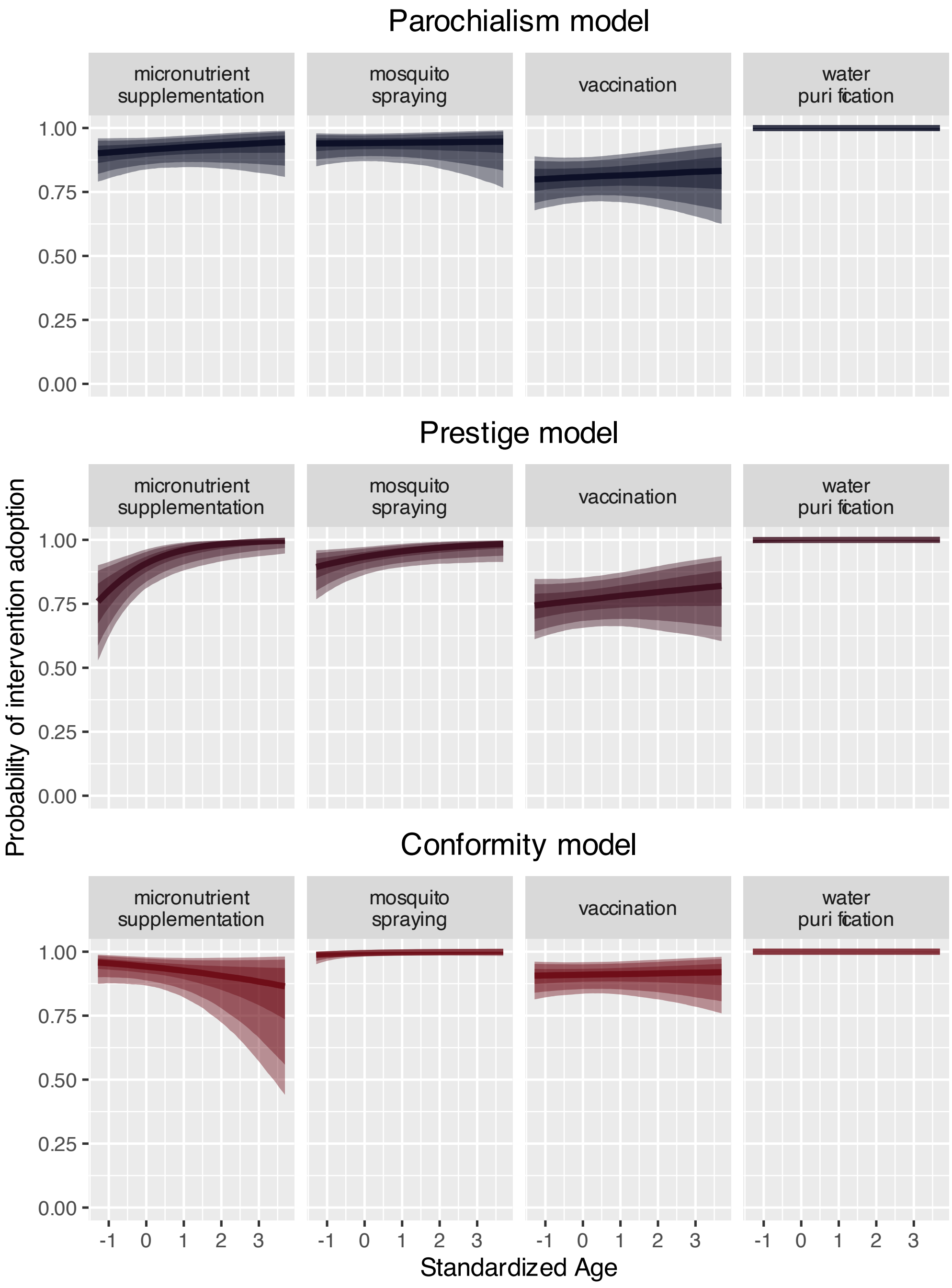

Figure S11: Posterior predictions for sex on intervention uptake by condition Point and whisker plots represent posterior median, and 66% (thick line) and 95% (think line) credible intervals.

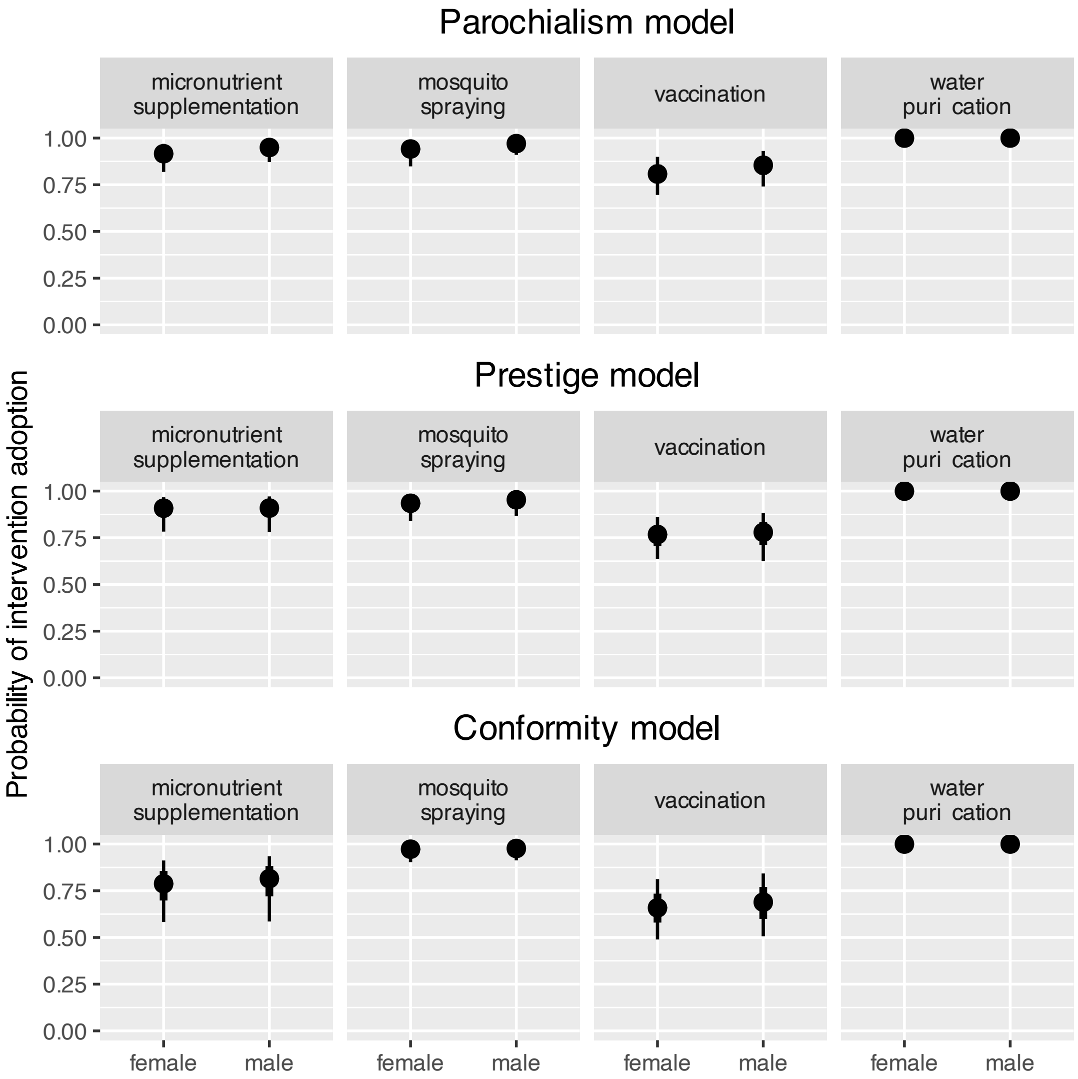

Figure S12: Difference in the posterior predictions by intervention advocate. Here we calculate and plot the differences in posterior predictions by intervention advocate for each intervention type.

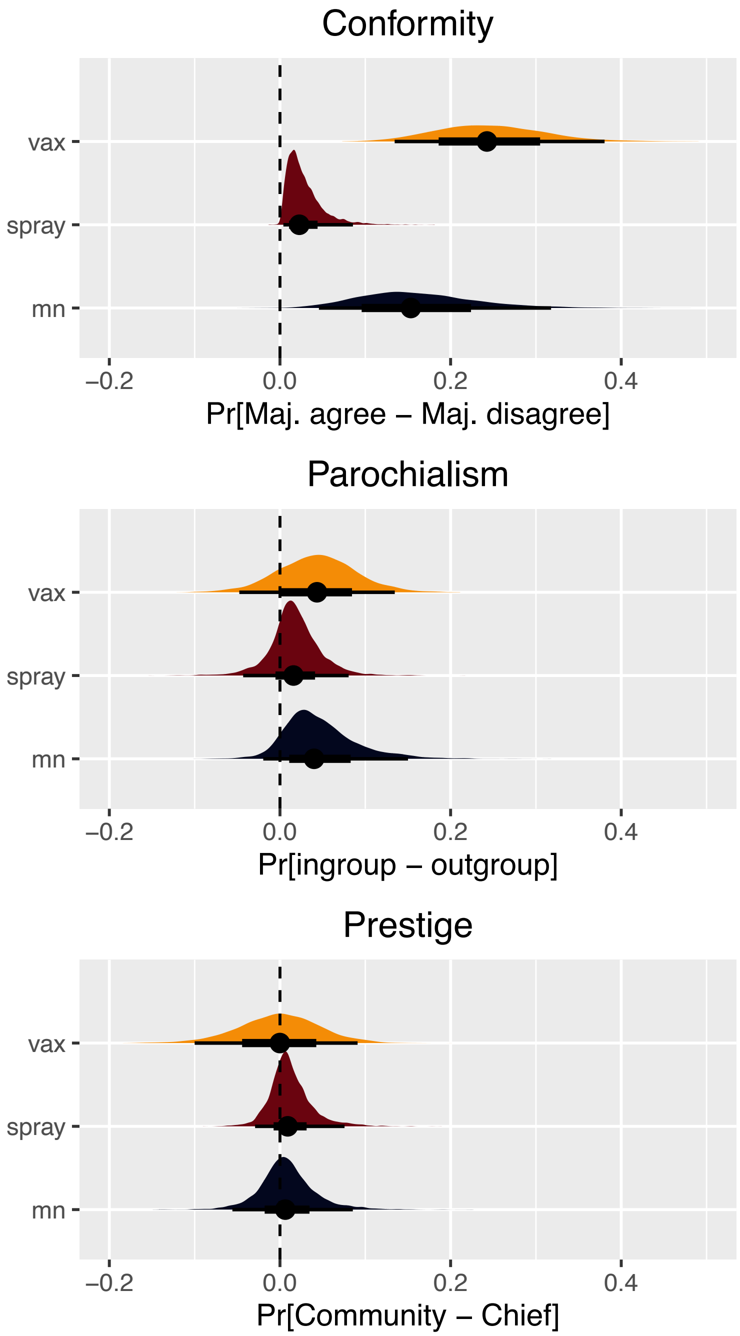

Citations

Bürkner, Paul-Christian. "Brms: An R Package for Bayesian Multilevel Models Using Stan." Journal of Statistical Software 80, no. 1 (2017): 1. https://doi.org/10.18637/jss.v080.i01.

Chalmers, R. Philip. "Mirt : A Multidimensional Item Response Theory Package for the R Environment." Journal of Statistical Software 48, no. 6 (2012). https://doi.org/10.18637/jss.v048.i06.

Scelza, Brooke A., Sean P. Prall, and Nancy E. Levine. "The Disequilibrium of Double Descent: Changing Inheritance Norms among Himba Pastoralists." Philosophical Transactions of the Royal Society B: Biological Sciences 374, no. 1780 (2019): 1780. https://doi.org/10.1098/rstb.2018.0072.
